# Long-term exposure to fine particulate matter constituents and incident stroke risk among middle-aged and older adults in China: a cohort study

**DOI:** 10.3389/fpubh.2026.1909901

**Published:** 2026-09-09

**Authors:** Hao Wang, Miaomiao Wei, Taikun Lu, Chao Wang, Xiaoshuang Xia, Lin Wang, Xin Li

**Affiliations:** 1 Department of Neurology, The Second Hospital of Tianjin Medical University, Tianjin, China; 2 Department of Neurosurgery, Peking Union Medical College Hospital, Chinese Academy of Medical Science and Peking Union Medical College, Beijing, China; 3 Department of Geriatrics, The Second Hospital of Tianjin Medical University, Tianjin, China; 4 Tianjin Health Meteorological Cross Innovation Center, Tianjin, China

**Keywords:** stroke, fine particulate matter constituents, CHARLS, long-term exposure, cohort study

## Abstract

**Background:**

Although long-term exposure to ambient fine particulate matter (PM_2.5_) is an established risk factor for stroke, evidence on the detrimental effects of its specific constituents remains limited. This study aimed to investigate the associations between long-term exposure to PM_2.5_ constituents and incident stroke, and to identify the key contributor.

**Methods:**

We conducted a nationwide prospective cohort study using data from the China Health and Retirement Longitudinal Study (CHARLS) between 2013 and 2018. Incident stroke cases were identified from CHARLS self-reported physician diagnoses during follow-up interviews. PM_2.5_ constituent concentration data were derived from the Tracking Air Pollution in China (TAP) dataset, while temperature and relative humidity data were obtained from the European Center for Medium-Range Weather Forecasts Reanalysis v5 (ERA5). Individual records were matched with environmental exposure data to quantify the associations between specific PM_2.5_ constituents (sulfate, nitrate, ammonium, organic matter, and black carbon) and the incident stroke. Multivariate Cox models and quantile-based g-computation were applied to assess the associations of PM_2.5_ constituents with incident stroke and to identify the key contributor.

**Results:**

Over a median follow-up of 5.0 years, 610 incident stroke cases occurred among 8,724 participants. All five constituents were significantly associated with stroke onset, the adjusted hazard ratios (95% confidence interval [CI]) for each interquartile range increase were 1.55 (95% CI: 1.35–1.79) for PM_2.5_, 1.55 (95% CI: 1.34–1.79) for sulfate, 1.37 (95% CI: 1.18–1.59) for nitrate, 1.42 (95% CI: 1.24–1.63) for ammonium, 1.59 (95% CI: 1.40–1.80) for organic matter, and 1.77 (95% CI: 1.54–2.03) for black carbon, respectively. In the joint effect analysis, black carbon (28.6%) exhibited the largest weight within the mixture. Stratified analyses revealed that individuals aged over 65 years and residents of southern China were more susceptible.

**Conclusion:**

Long-term exposure to PM_2.5_ constituents is associated with an elevated risk of incident stroke, with black carbon showing the strongest association among the five constituents. Older adults and residents of southern China were found to exhibit heightened susceptibility. These findings highlight the importance of mitigating the growing stroke burden through tailored clinical approaches and targeted regulation of carbonaceous emissions, accounting for demographic and regional vulnerabilities.

## 1 Introduction

Despite significant progress in treatment and prevention, stroke remains the second leading cause of death and a primary source of chronic disability in the world, a burden that falls disproportionately on aging populations. In 2021, stroke caused 7.3 million deaths and 160.5 million disability-adjusted life years (DALYs) worldwide (1). Pathophysiologically, stroke results from cerebral hemorrhage or obstruction and is clinically categorized into ischemic stroke, intracerebral hemorrhage, and subarachnoid hemorrhage (2). Previous compelling evidence indicates that around 90% of stroke cases can be attributed to modifiable risk factors, including air pollution, insufficient physical activity, obesity, unhealthy diet, and smoking (3, 4). Among these, air pollution is estimated to be responsible for 14% of stroke-related mortality (5). Fine particulate matter (PM_2.5_), defined as airborne particles with aerodynamic diameters ≤ 2.5 micrometers, represents a critical environmental pollutant. These microscopic particles demonstrate remarkable permeability through pulmonary alveolar membranes into systemic circulation, potentially inducing neurological damage (6).

Substantial evidence confirms that both short- and long-term exposure to PM_2.5_ is associated with an increased risk of stroke (7). Short-term exposure significantly links to a higher incidence, hospital admission rate, and mortality from stroke, with stronger associations observed for ischemic stroke and among females and older adults (8–10). Beyond these acute health effects, prospective studies in China indicate that long-term PM_2.5_ exposure is associated with an elevated risk of stroke incidence, mortality, and reduced survival, demonstrating robust associations for both ischemic and hemorrhagic subtypes (11–13). Such positive effects were also observed in Europe (14), Australia (15), and Africa (16), indicating that the association between PM_2.5_ exposure and stroke represents a global public health concern. Apart from its role in primary incidence, a study in the United States revealed that long-term PM_2.5_ exposure adversely affects stroke prognosis by increasing the risk of near-term hospital readmission (17). However, the existing literature is not entirely consistent, as some investigations have reported non-significant associations between PM_2.5_ and stroke-related hospitalizations or mortality (18–20). This heterogeneity may stem from differences in population characteristics, exposure assessment methods, or variations in the chemical composition of PM_2.5_ across different regions. This emphasizes the significance of pinpointing the specific toxicity of its individual components on stroke.

PM_2.5_ is a complex mixture composed primarily of sulfate, nitrate, ammonium, organic matter, and black carbon, originating from diverse sources like vehicular exhaust, industrial operations, and residential fuel combustion. Each constituent possesses distinct physicochemical and toxicological properties. Consequently, equal increments in PM_2.5_ mass concentration can lead to divergent health effects due to the source-specific composition. This underscores the necessity of evaluating the specific toxicity of a single component to formulate targeted and health-oriented emission control strategies.

Current epidemiological evidence on the associations between specific PM_2.5_ constituents and stroke remains both inconsistent and geographically limited. For instance, a case-crossover study in Boston discovered a connection between elevated black carbon levels and an increased risk of stroke incidence (21). Another time-series study in Guangzhou associated all five major PM_2.5_ constituents with stroke mortality (22). Nevertheless, findings from these single-city studies may lack generalizability. Moreover, few studies have examined the joint effects of exposure to multiple PM_2.5_ constituents or identified susceptible subpopulations within a nationwide cohort.

To address these knowledge gaps, we conducted a prospective nationwide cohort study in China. Our objectives were as follows: (1) to comprehensively examine the associations between long-term exposure to major PM_2.5_ constituents and first-ever stroke occurrence; (2) to evaluate their joint effects and individual contributions using a novel mixture modeling approach; and (3) to identify potential susceptible subpopulations and characterize exposure-response relationships. By moving beyond total PM_2.5_ mass, our study aims to provide a scientific basis for informing more precise and effective emission control strategies for stroke prevention.

## 2 Methods

### 2.1 Study design and population

**Figure 1:**
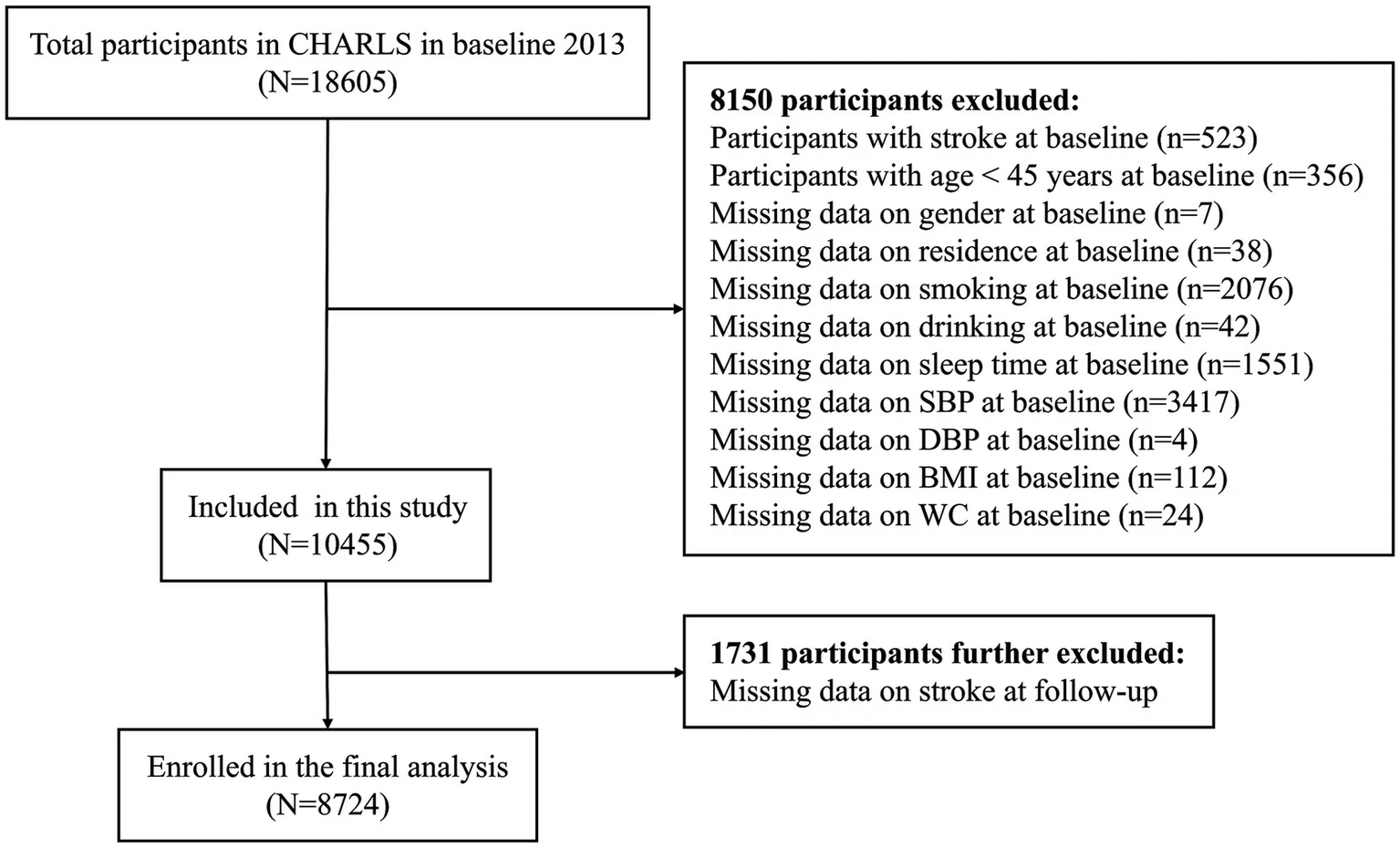
Flow chart of the study inclusion and exclusion criteria from the China Health and Retirement Longitudinal Study (CHARLS). Exclusions were performed sequentially, and numbers at each step represent additional exclusions from the remaining sample after prior exclusions had been removed. SBP, systolic blood pressure; DBP, diastolic blood pressure; BMI, body mass index; WC, waist circumference.

This prospective cohort study was conducted with China Health and Retirement Longitudinal Study (CHARLS)^1^ (23), an ongoing longitudinal study of middle-aged and older Chinese adults. As a nationally representative survey, CHARLS encompasses participants from 450 communities across 126 cities and counties in 28 provinces in China. Our baseline was established in 2013, coinciding with the implementation of China’s Air Pollution Prevention and Control Action Plan (APPCAP), enabling the examination of associations within a contemporary context of declining air pollution. It is estimated that, compared with the levels in 2013, the annual average concentrations of PM_2.5_ in 2017 witnessed a 33.3% decline in 74 key Chinese cities (24). We restricted follow-up to 2018 and excluded the 2020 wave to avoid potential confounding from the COVID-19 pandemic, which disrupted healthcare access and stroke diagnosis. Consequently, 18,605 participants were enrolled initially and a total of 10,455 participants met the inclusion criteria. At baseline, they had no history of stroke, were 45 years or older, and had complete demographic, health, and physical examination data. Participants were followed up in 2015 and 2018 via face-to-face, computer-assisted personal interviews to track the same survey as in 2013. After excluding 1731 participants due to missing follow-up, 8,724 participants were included in the final analysis. Exclusions were performed sequentially, with each step applied to the remaining participants after prior exclusions had been removed. The clear participant selection process is presented in Figure 1.

All participants recruited into the CHARLS program were given written informed consent. The CHARLS adheres strictly to the ethical principles outlined in the Declaration of Helsinki. Ethical approval for the study protocol and data collection procedures was obtained from the Ethics Review Committee of Peking University (IRB00001052-11015).

### 2.2 Assessment of incident stroke

The CHARLS public-use dataset does not provide ICD-10 diagnostic codes, individual-level hospital medical records, or external cause-of-death registries. Incident stroke cases were identified based on participants’ responses to specific survey questions regarding a first-ever physician diagnosis during follow-up interviews. This included information from primary care, self-reported data, and hospital admissions. The timing of the stroke event was ascertained by asking participants to report either the year or their age at the time of occurrence. Participants were followed from the 2013 baseline across three interview waves until the time of stroke diagnosis, loss of contact (16.6%), or until the survey end in 2018, whichever occurred first.

### 2.3 Environmental exposure assessment

The daily concentrations of PM_2.5_ and its key constituents, including sulfate, nitrate, ammonium, organic matter and black carbon during the study period were retrieved from Tracking Air Pollution in China (TAP) dataset^2^. The TAP dataset provides high-resolution estimates by integrating the Weather Research and Forecasting-Community Multiscale Air Quality (WRF-CMAQ) modeling system, ground-based observations, a machine learning-extreme gradient boosting algorithm, and multisource satellite-based PM_2.5_ data, yielding daily predictions at a 10 × 10 km spatial resolution across China (25, 26). Validation studies have demonstrated strong correlations between TAP estimates and in-situ measurements, with correlation coefficients ranging from 0.67 to 0.80 on a daily scale (2013–2020) and from 0.64 to 0.75 on a monthly scale (2000–2020) (26). Daily temperature and relative humidity data for the same period were extracted from the European Center for Medium-Range Weather Forecasts Reanalysis v5 (ERA5) of the global climate^3^ (27). The ERA5 has a spatial resolution of 0.1° × 0.1° and a temporal resolution of 1 h. All participants in the CHARLS cohort were geo-coded to the regionalization code of their residential address to uphold participant privacy. Consequently, the daily gridded PM_2.5_ constituent concentrations were initially aggregated to city-level averages, which were used for monthly average calculations. For each participant, long-term exposure to PM_2.5_ constituents was defined as the cumulative average concentration from the baseline interview month to the endpoint (28). For participants who experienced a stroke event during follow-up, exposure was calculated as the average concentration from baseline to the month of the event. For those without an event, exposure was averaged from baseline to the end of follow-up. Daily temperature and relative humidity data from the ERA5 were processed using the same city-level aggregation and the same average approach.

### 2.4 Covariates

Potential confounders of the associations between pollutant exposure and incident stroke were selected from the CHARLS questionnaire based on previous investigations (29, 30). The analyses incorporated covariables from sociodemographic characteristics, health-related behaviors, and anthropometric measurements. These covariables encompassed age, gender (female vs. male), education (“primary school or below”, “high school”, and “college or above”), marital status (married vs. unmarried), residence (rural vs. urban), smoking (no vs. yes), drinking (no vs. yes), sleep time (self-reported hours per night), social activity (no vs. yes), hypertension (no vs. yes), dyslipidemia (no vs. yes), diabetes (no vs. yes), heart disease (including myocardial infarction, coronary heart disease, angina, congestive heart failure, or other heart problems) (no vs. yes), systolic blood pressure (SBP, average of three measurements), diastolic blood pressure (DBP, average of three measurements), body mass index (BMI, calculated as weight in kilograms divided by height in meters squared), waist circumference (WC, measured in centimeters). All of these variables, in combination with temperature and relative humidity, were employed to exhibit characteristics of participants.

### 2.5 Statistical analysis

Statistical analyses and result visualizations were performed using R software (version 4.4.1, R Core Team). Statistical significance was defined as a two-tailed *p*-value < 0.05. Extreme values in continuous variables were handled by winsorizing at the 1st and 99th percentiles. We compared the characteristics of the participants by stratification of incident stroke. Continuous variables were presented as the mean ± standard deviation (SD), while categorical variables were expressed as frequencies (%). *T*-tests were employed to analyze continuous variables (such as age, sleep time, and BMI), and chi-square tests were used to examine categorical variables (such as gender, marital status, and hypertension). Pearson correlation analysis was conducted to examine the interrelationships between PM_2.5_ and its constituents. Variance inflation factors (VIF) were calculated to detect multicollinearity, with VIF ≥ 10 indicating serious multicollinearity.

Multivariate Cox models were used to quantify the association between PM_2.5_ constituents and incident stroke. Given the potential multicollinearity among PM_2.5_ constituents, we estimated the hazard ratios (HRs) and 95% confidence intervals (CIs) per interquartile range (IQR) increase in each constituent using single-pollutant models. Model 1 did not incorporate any covariates. Model 2 was adjusted for demographic characteristics (age, gender, education, marital status, residence). In model 3, we further adjusted for lifestyle factors (smoking, drinking, sleep time, social activity), clinical conditions (hypertension, dyslipidemia, diabetes, heart disease), anthropometric measures (BMI, WC), and environmental factors (temperature, relative humidity) in addition to the covariates in model 2. To examine possible nonlinear relationships between fine particulate constituents and stroke incidence, exposure-response curves were examined using the restricted cubic spline (RCS) models with three knots at the 10th, 50th, and 90th percentiles. The reference value was set at the median concentration of each pollutant. Analyses were restricted to the 5th-95th percentile range of exposures, and likelihood ratio tests were used to assess nonlinearity. To determine the concentration threshold at which the incident stroke risk began to increase markedly, we applied segmented regression models. The breakpoint in each model, marking the concentration of most significant slope change, was defined as the threshold.

To assess the joint effects of simultaneous exposure to PM_2.5_ constituents, we employed quantile-based g-computation (QGC) to evaluate the overall mixture effect of five PM_2.5_ constituents on incident stroke risk. The QGC model is a parametric statistical method that combines weighted quantile sum (WQS) regression with the flexibility of g-computation (31). In our QGC analysis, the five constituents were divided into quartiles (*q* = 4) to balance model flexibility with statistical power. The QGC model estimated the joint effect of increasing all constituents simultaneously by one quartile, presented as the HR with a 95% CI. Compared to WQS regression, QGC offers several advantages that make it particularly suitable for analyzing highly correlated pollutant mixtures: (1) it relaxes the unidirectional assumption, allowing constituents to have either positive or negative weights; (2) it provides more robust estimation in the presence of severe multicollinearity; (3) it better adapts to nonlinear exposure-response relationships observed in our preliminary analyses. Component weights were calculated to show the relative contribution of each constituent to the overall mixture effect, with weights summing to 1 in absolute value. The WQS analysis was conducted as a sensitivity analysis using three quantiles (*q* = 3) and bootstrap resampling (*b* = 100), maintaining the unidirectional positive effect assumption.

To identify potential susceptible subpopulations, we performed stratified analyses according to age (< 65 vs. ≥ 65 years), gender, education level (primary school or below vs. high school or above), marital status, geographic region (North vs. South China), smoking status, and BMI (< 24 vs. ≥ 24 kg/m^2^). Educational attainment was divided into two categories to balance the sizes of the groups and ensure statistical robustness. Geographic stratification was based on the Qinling Mountains-Huai River line, a well-recognized boundary that reflects distinct climatic and environmental gradients in China. This regional classification, consistent with prior research (32), was derived from participants’ geolocation data. Since this was a post-hoc derived variable that was not collected during the baseline assessment, it was excluded from the baseline characteristics. BMI was categorized using the Chinese criteria recommended by the Working Group on Obesity in China (WGOC), with <24 kg/m^2^ defined as non-overweight and ≥24 kg/m^2^ as overweight. Interaction terms between each PM_2.5_ constituent and subgroup variables were tested using likelihood ratio tests to assess statistical significance of effect modification.

Comprehensive sensitivity analyses were conducted to test the robustness of our findings: (1) Residual-based toxicity ranking validation: to verify the stability of the identified toxicity hierarchy independent of total PM_2.5_ mass, we calculated residuals for each constituent from linear regression models with total PM_2.5_ mass as the independent variable. These residuals represent constituent-specific variations orthogonal to total mass. We then applied Cox proportional hazards models to these residual values to re-assess the relative toxicity weights; (2) extreme value exclusion: we re-analyzed the associations after excluding extreme exposure values (beyond the 1st and 99th percentiles) for each PM_2.5_ constituent to assess the influence of potential outliers on effect estimates; (3) proportional hazards assumption assessment: we systematically evaluated the proportional hazards assumption using Schoenfeld residuals tests. For constituents violating this assumption (global test *p* < 0.05), we employed stratified Cox models by gender to accommodate time-varying hazard ratios. All sensitivity analyses maintained the same covariate adjustment as in model 3 and expressed effect sizes as HRs per IQR increase to ensure comparability with primary results.

## 3 Results

### 3.1 Characteristics of study participants and exposure distributions

As shown in Table 1, the characteristics were compared between the incident stroke and non-stroke populations. A total of 8,724 participants (mean age 59.95 ± 8.86 years; 49.16% male) were included in this prospective cohort study. During follow-up, 610 individuals (7.0%) developed stroke. Compared with the non-stroke group, participants who experienced stroke were significantly older, more likely to be unmarried and current smokers, had shorter sleep duration, and exhibited higher systolic and diastolic blood pressure, BMI, and WC. They also had significantly higher prevalence rates of hypertension (49.18% vs. 24.90%), dyslipidemia (21.80% vs. 10.67%), diabetes (12.95% vs. 6.51%), and heart disease (23.44% vs. 12.24%). A comparison of baseline characteristics between participants included in the final analysis and those excluded due to missing data or loss to follow-up is provided in Supplementary Table S1. In terms of environmental exposure, stroke cases were associated with significantly lower ambient temperature and relative humidity. The average concentrations of PM_2.5_ total mass, sulfate, nitrate, ammonium, organic matter and black carbon during the study period were 50.8, 9.3, 10.8, 7.3, 12.4, and 2.4 μg/m^3^, respectively (Table 2). Distributions of PM_2.5_ constituents stratified by residence are presented in Supplementary Tables S2, S3. The spatial distributions of ambient PM_2.5_ and its five constituents across China are presented in Supplementary Figure S1. Strong positive correlations were observed between PM_2.5_ and its constituents, as well as among the constituents themselves (Pearson correlation coefficient r > 0.95; Supplementary Figure S2). Consequently, VIF analysis revealed severe multicollinearity, with all VIFs substantially exceeding 10 (Supplementary Figure S3).

**Table 1:** The characteristics of participants by incident stroke status during follow-up.

Characteristics	Overall (*n* = 8,724)	Non-stroke (*n* = 8,114)	Stroke (*n* = 610)	*p*-value
Age (years)	59.95 ± 8.86	59.75 ± 8.86	62.56 ± 8.46	< 0.001
Gender, *n* (%)				0.290
Female	4,435 (50.84)	4,138 (51.00)	297 (48.69)	
Male	4,289 (49.16)	3,976 (49.00)	313 (51.31)	
Education, *n* (%)				0.868
Primary school or below	7,792 (89.32)	7,248 (89.33)	544 (89.18)	
High school	799 (9.16)	741 (9.13)	58 (9.51)	
College or above	133 (1.52)	125 (1.54)	8 (1.31)	
Marital status, *n* (%)				0.013
Married	7,727 (88.57)	7,206 (88.81)	521 (85.41)	
Unmarried	997 (11.43)	908 (11.19)	89 (14.59)	
Residence, *n* (%)				0.153
Rural	7,145 (81.90)	6,659 (82.07)	486 (79.67)	
Urban	1,579 (18.10)	1,455 (17.93)	124 (20.33)	
Smoking, *n* (%)				0.003
No	4,793 (54.94)	4,493 (55.37)	300 (49.18)	
Yes	3,931 (45.06)	3,621 (44.63)	310 (50.82)	
Drinking, *n* (%)				0.574
No	4,665 (53.47)	4,346 (53.56)	319 (52.30)	
Yes	4,059 (46.53)	3,768 (46.44)	291 (47.70)	
Sleep time (hours)	6.18 ± 1.82	6.19 ± 1.80	6.03 ± 2.03	0.043
Social activity, *n* (%)				0.728
No	3,555 (40.75)	3,311 (40.81)	244 (40.00)	
Yes	5,169 (59.25)	4,803 (59.19)	366 (60.00)	
Hypertension, *n* (%)				< 0.001
No	6,404 (73.41)	6,094 (75.10)	310 (50.82)	
Yes	2,320 (26.59)	2020 (24.90)	300 (49.18)	
Dyslipidemia, *n* (%)				< 0.001
No	7,725 (88.55)	7,248 (89.33)	477 (78.20)	
Yes	999 (11.45)	866 (10.67)	133 (21.80)	
Diabetes, *n* (%)				< 0.001
No	8,117 (93.04)	7,586 (93.49)	531 (87.05)	
Yes	607 (6.96)	528 (6.51)	79 (12.95)	
Heart disease, *n* (%)				< 0.001
No	7,588 (86.98)	7,121 (87.76)	467 (76.56)	
Yes	1,136 (13.02)	993 (12.24)	143 (23.44)	
SBP (mmHg)	129.89 ± 20.72	129.24 ± 20.44	138.50 ± 22.35	< 0.001
DBP (mmHg)	76.48 ± 13.11	76.20 ± 13.02	80.28 ± 13.71	< 0.001
BMI (kg/m^2^)	23.84 ± 3.63	23.78 ± 3.61	24.53 ± 3.73	< 0.001
WC (cm)	86.71 ± 10.91	86.50 ± 10.85	89.48 ± 11.29	< 0.001
Temperature (°C)	15.76 ± 4.67	15.90 ± 4.57	13.88 ± 5.44	< 0.001
Relative humidity (%)	74.69 ± 10.12	74.87 ± 10.13	72.42 ± 9.75	< 0.001

SBP, systolic blood pressure; DBP, diastolic blood pressure; BMI, body mass index; WC, waist circumference.

**Table 2:** The distributions of PM_2.5_ and its constituents during the study period.

Variables	Mean	SD	Minimum	P_25_	Median	P_75_	Maximum
PM_2.5_ (μg/m^3^)	50.8	18.3	17.2	35.6	49.1	63.3	118.1
Sulfate (μg/m^3^)	9.3	3.0	3.4	6.6	9.3	11.8	19.7
Nitrate (μg/m^3^)	10.8	4.8	2.5	6.7	11.0	14.9	26.5
Ammonium (μg/m^3^)	7.3	2.9	2.2	4.8	7.5	9.5	16.7
Organic matter (μg/m^3^)	12.4	4.1	4.5	9.2	11.8	14.8	35.7
Black carbon (μg/m^3^)	2.4	0.7	1.1	1.9	2.3	2.9	5.7

SD, standard deviation; P_25_, 25th percentile (first quartile); P_75_, 75th percentile (third quartile); PM_2.5_, particulate matter with aerodynamic diameter ≤ 2.5 μm.

### 3.2 Associations of PM2.5 constituents with stroke incidence

**Figure 2:**
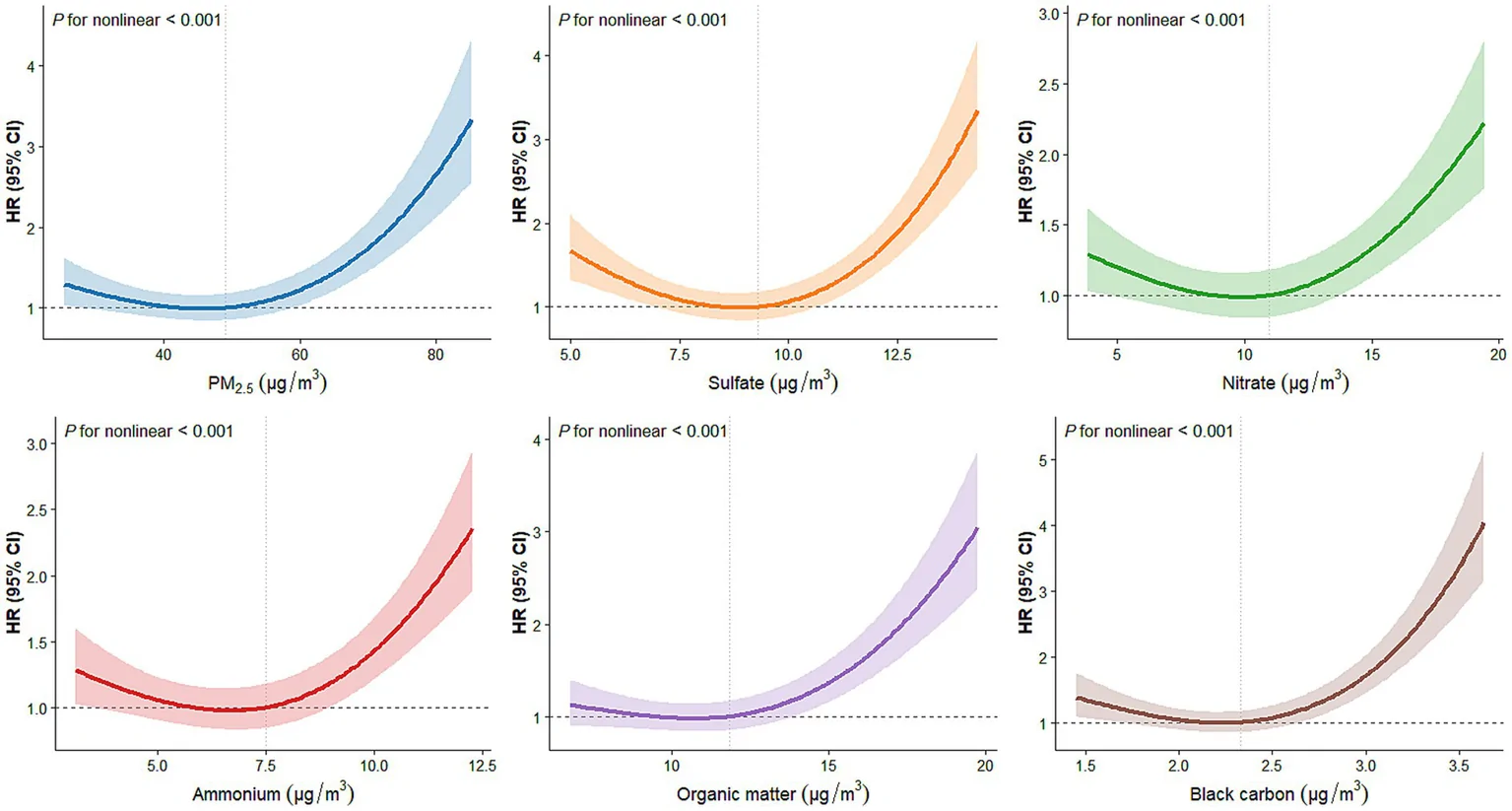
Exposure-response relationships of PM_2.5_ and its constituents with the risk of incident stroke. The model was adjusted for age, gender, education, marital status, residence, smoking, drinking, sleep time, social activity, hypertension, dyslipidemia, diabetes, heart disease, body mass index, waist circumference, temperature, and relative humidity. HR, hazard ratio; CI, confidence interval; PM_2.5_, particulate matter with aerodynamic diameter ≤ 2.5 μm.

During a median follow-up of 5.0 years (range: 0.17–5.25 years), corresponding to 42,354 person-years of observation, the stroke incidence rate was 14.4 per 1,000 person-years. In the single-pollutant Cox models, each IQR increase in PM_2.5_ and its constituents was significantly associated with a higher risk of incident stroke across all three models, with all *p*-values < 0.001 (Table 3). In the fully adjusted model (model 3), the HRs were highest for black carbon (HR = 1.77, 95% CI: 1.54–2.03), followed by organic matter (HR = 1.59, 95% CI: 1.40–1.80), PM_2.5_ total mass (HR = 1.55, 95% CI: 1.35–1.79), sulfate (HR = 1.55, 95% CI: 1.34–1.79), ammonium (HR = 1.42, 95% CI: 1.24–1.63), and nitrate (HR = 1.37, 95% CI: 1.18–1.59). The exposure-response relationships of PM_2.5_ and its constituents with stroke incidence were all significantly non-linear (all *p* for nonlinearity < 0.001), exhibiting U-shaped patterns as depicted in Figure 2. Detailed metrics of these curves are provided in Supplementary Table S4. Notably, black carbon demonstrated the most pronounced health risk, characterized by the highest maximum HR (4.04), and the lowest concentration threshold (2.8 μg/m^3^).

**Table 3:** Association of PM_2.5_ and its constituents with the risk of incident stroke by the single-pollutant model.

Pollutants	IQR	Model 1^a^ HR (95% CI)	*p*-value	Model 2^b^ HR (95% CI)	*p*-value	Model 3^c^ HR (95% CI)	*p*-value
PM_2.5_	27.7	1.49 (1.32–1.68)	< 0.001	1.50 (1.33–1.69)	< 0.001	1.55 (1.35–1.79)	< 0.001
Sulfate	5.1	1.48 (1.29–1.69)	< 0.001	1.47 (1.28–1.69)	< 0.001	1.55 (1.34–1.79)	< 0.001
Nitrate	8.3	1.43 (1.25–1.64)	< 0.001	1.43 (1.25–1.64)	< 0.001	1.37 (1.18–1.59)	< 0.001
Ammonium	4.7	1.43 (1.26–1.63)	< 0.001	1.43 (1.26–1.63)	< 0.001	1.42 (1.24–1.63)	< 0.001
Organic matter	5.6	1.44 (1.30–1.60)	< 0.001	1.45 (1.30–1.61)	< 0.001	1.59 (1.40–1.80)	< 0.001
Black carbon	1.0	1.54 (1.37–1.73)	< 0.001	1.55 (1.38–1.74)	< 0.001	1.77 (1.54–2.03)	< 0.001

IQR, interquartile range; HR, hazard ratio; CI, confidence interval; PM_2.5_, particulate matter with aerodynamic diameter ≤ 2.5 μm.

a Model 1 did not incorporate any covariates.

b Model 2 was adjusted for age, gender, education, marital status and residence.

c Model 3 was adjusted for age, gender, education, marital status, residence, smoking, drinking, sleep time, social activity, hypertension, dyslipidemia, diabetes, heart disease, body mass index, waist circumference, temperature, and relative humidity.

### 3.3 Joint effects of PM2.5 constituent mixtures

**Figure 3:**
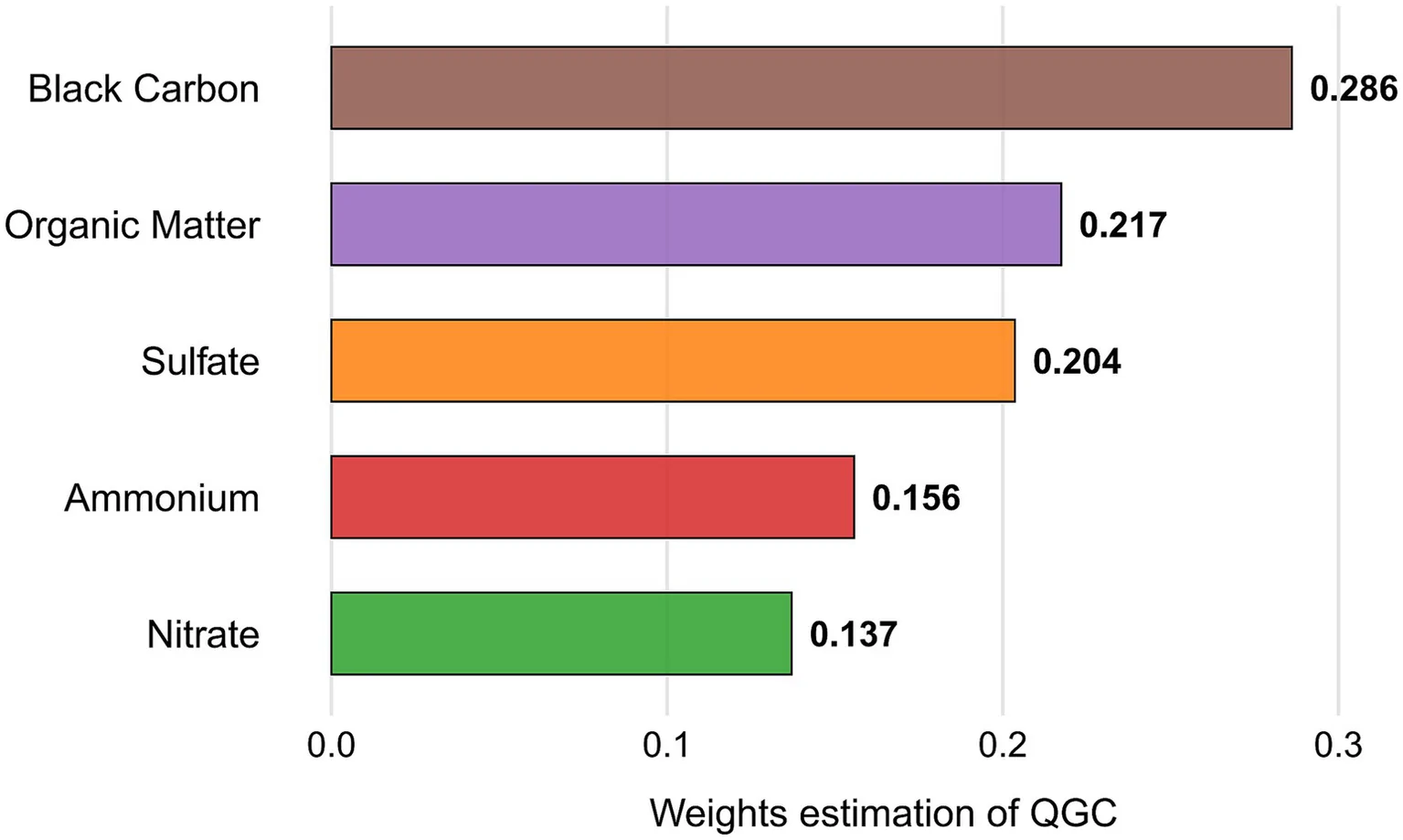
Weights of PM_2.5_ constituents in the mixture index for the association between the particle mixture and incident stroke by QGC. The model was adjusted for age, gender, education, marital status, residence, smoking, drinking, sleep time, social activity, hypertension, dyslipidemia, diabetes, heart disease, body mass index, waist circumference, temperature, and relative humidity. PM_2.5_, particulate matter with aerodynamic diameter ≤ 2.5 μm; QGC, quantile-based g-computation.

The results from the QGC analysis are presented in Figure 3. We observed a significant positive association between the joint exposure to the PM_2.5_ mixture and incident stroke. Each one-quantile increase in the overall mixture was associated with a 20.90% higher risk of stroke (HR = 1.21, 95% CI: 1.11–1.31). When scaled per IQR increase, the risk was substantially elevated by 46.10% (HR = 1.46, 95% CI: 1.24–1.72). Among all constituents, black carbon was identified as the largest contributor in the QGC model, accounting for the largest weight (28.61%), followed by organic matter (21.70%) and sulfate (20.40%).

### 3.4 Stratified analyses

The stratified analyses included the following subgroup sizes: age <65 years (*n* = 6,198) and ≥65 years (*n* = 2,526); male (*n* = 4,289) and female (*n* = 4,435); primary school or below (*n* = 7,792) and high school or above (*n* = 932); married (*n* = 7,727) and unmarried (*n* = 997); Southern China (*n* = 4,691) and Northern China (*n* = 4,033); non-smokers (*n* = 4,793) and smokers (*n* = 3,931); and BMI < 24 kg/m^2^ (*n* = 4,801) and ≥24 kg/m^2^ (*n* = 3,923).

**Figure 4:**
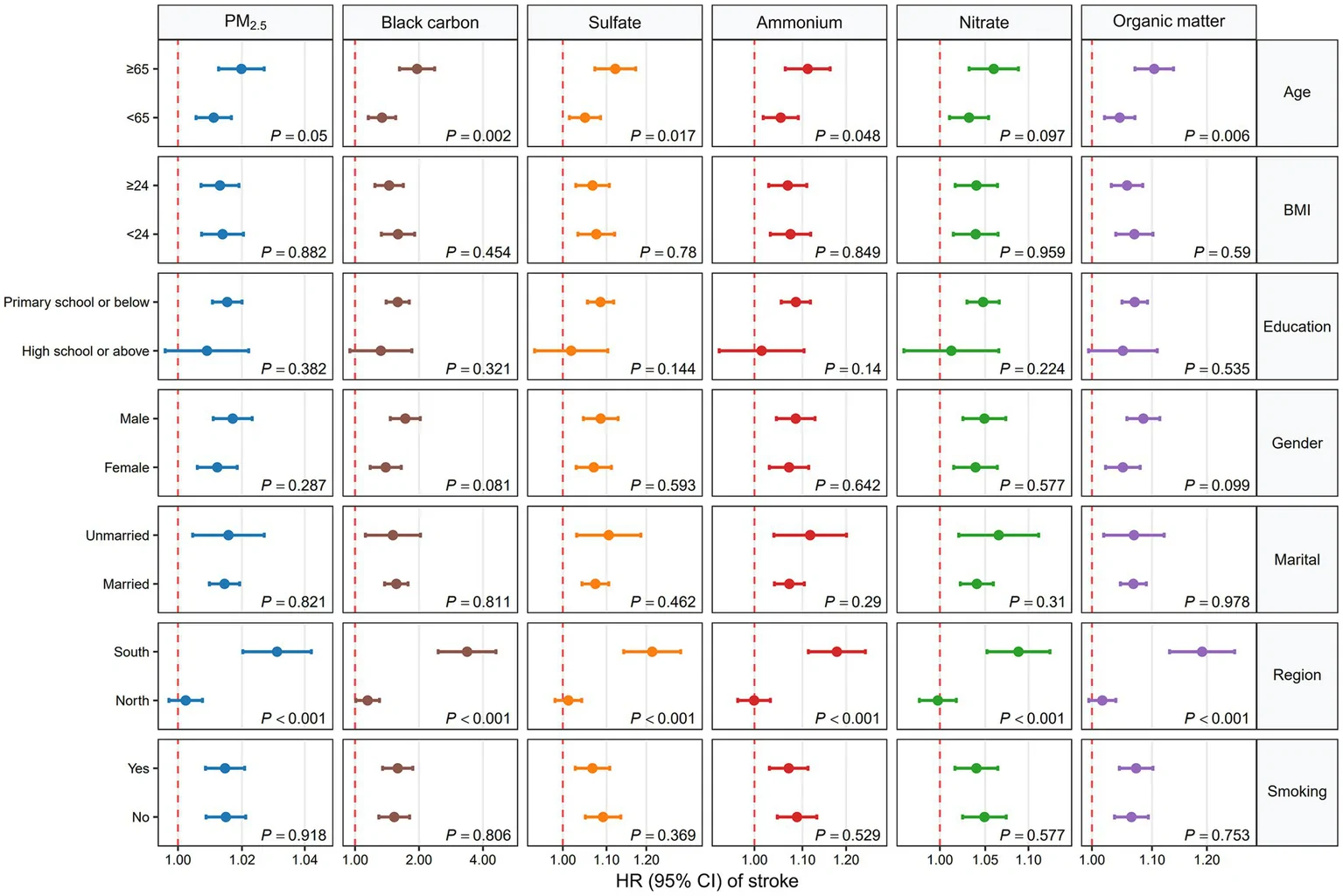
Hazard ratio (HR) (95% confidence interval [CI]) for stroke incidence associated with per interquartile range increase in PM_2.5_ and its constituents, stratified by age, BMI, education, gender, marital status, geographic region, and smoking status. Within each stratum, models were adjusted for all covariates except the stratification variable. The adjustment set included: Age, gender, education, marital status, residence, smoking, drinking, sleep time, social activity, hypertension, dyslipidemia, diabetes, heart disease, body mass index, waist circumference, temperature, and relative humidity. The *p* values shown in the figure are for the test of interaction between each pollutant and the stratification variables. PM_2.5_, particulate matter with aerodynamic diameter ≤ 2.5 μm; BMI, body mass index; HR, hazard ratio; CI, confidence interval.

Stratified analyses revealed significant effect modifications by age and geographic region on the associations between PM_2.5_ and its constituents with incident stroke risk (Figure 4). The adverse effects of PM_2.5_ and its constituents were consistently stronger among older adults (≥ 65 years) compared to their younger counterparts (< 65 years). These differences were statistically significant for black carbon (*p* for interaction = 0.002), organic matter (*p* for interaction = 0.006), sulfate (*p* for interaction = 0.017), and ammonium (*p* for interaction = 0.048). A pronounced North–South geographic disparity was also observed. Residents of Southern China exhibited significantly heightened vulnerability to all pollutants assessed (all *p* for interaction < 0.001), with particularly strong associations for black carbon (HR = 3.00, 95% CI: 2.17–4.15 in the South vs. HR = 1.18, 95% CI: 1.04–1.34 in the North). In contrast, no statistically significant effect modification was detected for gender, BMI, education, marital status, or smoking status (all *p* for interaction > 0.05).

### 3.5 Sensitivity analyses

Sensitivity analyses confirmed the robustness of our primary findings. First, residual-based analysis robustly validated the identified toxicity hierarchy. When examining constituent-specific variations independent of total PM_2.5_ mass, black carbon consistently showed the largest effect estimate with HR of 6.84 (95% CI: 3.95–12.15) per IQR increase (1.0 μg/m^3^), accounting for 81.0% of the quantified toxicity. The identical ranking—black carbon > organic matter > sulfate > ammonium > nitrate—between residual-based and original QGC analyses demonstrates the stability of our findings against multicollinearity concerns (Supplementary Table S5). Second, results were consistent in a WQS regression, which also identified black carbon as the largest contributor to the mixture effect (Weight: 0.192, Supplementary Figure S4). Furthermore, the associations remained stable across different adjustment models in Cox regression (Supplementary Figure S5), persisted after excluding extreme exposure values (Supplementary Table S6), and remained consistent after addressing violations of the proportional hazards assumption (Supplementary Table S7). Gender-stratified analyses (Supplementary Table S8) indicated that while associations were generally positive in both genders, the risk estimates appeared higher for black carbon and organic matter in males, and for sulfate, ammonium, and nitrate in females.

## 4 Discussion

This prospective, nationwide cohort study provides evidence that long-term exposure to specific PM_2.5_ constituents (sulfate, nitrate, ammonium, organic matter, and black carbon) is significantly associated with an elevated risk of incident stroke among middle-aged and older Chinese adults. Among the five PM_2.5_ constituents, black carbon exhibited the strongest association with stroke incidence, followed by organic matter and sulfate. These adverse effects were more pronounced in adults over 65 and residents of southern China. To our knowledge, this is the first nationwide prospective cohort study in China to comprehensively assess the single and joint effects of specific PM_2.5_ constituents on incident stroke. Our findings thereby offer valuable evidence to guide the formulation of targeted emission control strategies and public health interventions for stroke prevention.

Our findings align with and extend the previous nationwide investigations on the stroke burden attributable to PM_2.5_ constituents. A time-stratified case-crossover study identified positive associations between short-term exposure to specific PM_2.5_ components and ischemic stroke hospital admissions (33), with excess risks of 1.77% (95% CI: 0.65–2.90%) for ammonium and 1.69% (95% CI: 0.99–2.39%) for indeno (1, 2, 3-cd) pyrene—a polycyclic aromatic hydrocarbon (PAH) within organic matter. For long-term effects on stroke mortality, a nationwide prospective cohort established significantly increased risks for all five major PM_2.5_ constituents (34), with HRs in descending order of magnitude as follows: black carbon (HR = 1.19, 95% CI: 1.07–1.38), nitrate (HR = 1.18, 95% CI: 1.04–1.42), ammonium (HR = 1.18, 95% CI: 1.04–1.41), organic matter (HR = 1.15, 95% CI: 1.05–1.30), and sulfate (HR = 1.14, 95% CI: 1.01–1.34). These results collectively reinforce the association between multiple PM_2.5_ constituents and stroke risk. Our study significantly advances this field by moving beyond single-pollutant models to dissect the complex mixture effects of PM_2.5_ constituents. The QGC model identified black carbon as the largest contributor to the joint mixture effect, followed by organic matter and sulfate. This hierarchy is consistent with findings from a large-scale inpatient study on stroke fatality (35), which identified the same three components as leading contributors based on population attributable fractions: black carbon (10.6%), organic matter (9.9%), and sulfate (6.6%). The robust association of black carbon was further corroborated in our residual-based analysis, where its effect persisted and intensified after accounting for total PM_2.5_ mass. This highlights a critical limitation of regulating PM_2.5_ solely by mass concentration and suggests that targeting specific constituents may be warranted.

Furthermore, we observed significant nonlinear exposure-response relationships for all constituents, characterized by U-shaped patterns. Several factors may contribute to this shape. First, the elevated risk at the lower tail of the exposure distribution could be partially attributable to residual confounding by unmeasured or imperfectly measured factors, such as socioeconomic status, dietary patterns, or healthcare access, which may correlate with both lower pollution exposure and higher stroke risk. Second, despite restricting our analyses to the 5th–95th percentile exposure range, statistical instability due to limited sample size at the extreme low-concentration end cannot be entirely excluded. Third, while speculative, biological mechanisms might also play a role, although this remains an open question requiring further investigation. Importantly, the apparent dip below unity at extremely low concentrations should not be interpreted as a protective effect of air pollution, but rather as a statistical artifact or a reflection of residual confounding. Whereas the prior inpatient study reported monotonically increasing risk curves (35), our analyses demonstrated that the risk increased progressively above specific thresholds. This pattern finds some support in earlier research (28), which suggested a potential plateauing of risk at lower concentrations. However, a pivotal finding from our RCS curves is that these thresholds were consistently low, particularly for black carbon (2.8 μg/m^3^), and the risk escalated sharply thereafter. This indicates that the observed risk of incident stroke rises appreciably at concentrations exceeding these low thresholds. This also suggests that moderate reductions in ambient levels of key pollutants, especially black carbon, could contribute to benefits for primary stroke prevention. Collectively, these considerations suggest that the observed U-shaped patterns are likely driven by methodological artifacts and residual confounding rather than biological protection, particularly at the lower end of the exposure distribution.

Our findings indicated black carbon and organic matter as the two major contributors in the mixture analysis, jointly accounting for over 50% of the quantified weight in the joint effect. In China, urban black carbon concentrations typically range from 1.0 to 7.5 μg/m^3^, considerably exceeding levels in most developed regions (36). Black carbon originates predominantly from incomplete combustion processes, including vehicular emissions, residential solid fuel burning, and industrial activities (37). Toxicologically, black carbon acts as a primary carrier of co-pollutants. Its large specific surface area facilitates the adsorption of various toxic substances, such as PAHs and heavy metals, thereby promoting synergistic effects that exacerbate oxidative stress and systemic inflammation (38, 39). This is consistent with findings that lead, selenium, and cadmium in PM_2.5_ related to coal combustion were associated with ischemic stroke (40). Additionally, black carbon has been shown to induce reactive oxygen species (ROS) generation, disrupt cellular calcium homeostasis, and cause mitochondrial dysfunction (41). Organic matter, which comprises a complex mixture of numerous compounds, stems from both primary combustion emissions and secondary atmospheric reactions. In addition to driving oxidative and antioxidative imbalances, certain organic matter components, particularly PAHs, are capable of binding to DNA, an interaction that may lead to mutagenic and carcinogenic outcomes (42). Collectively, these pathophysiological processes, combined with effects from other PM_2.5_ constituents, can induce a cascade of cerebrovascular damage, including increased blood coagulability, plaque destabilization, accelerated atherosclerosis, and sustained vascular inflammation, all of which may contribute to the risk of stroke onset (10, 43, 44).

The association between black carbon from traffic-related diesel combustion and stroke risk is supported by multiple lines of evidence. Short-term exposure has been shown to trigger the onset of large-artery atherosclerotic ischemic stroke (45), while long-term exposure has been associated with increased stroke incidence (46) and subclinical precursors like increased carotid intima-media thickness (47). Notably, the large-scale ELAPSE study further confirmed that the adverse effect of black carbon on stroke incidence persists even at low concentrations, underscoring its potency as a cerebrovascular risk factor even in relatively clean environments (48). Beyond black carbon, organic matter has also emerged as a component of concern, particularly for impairing neurological recovery after stroke (30). Furthermore, the health effects of air pollution can be modified by meteorological conditions. A nationwide study in China demonstrated that heat waves interacted synergistically with PM_2.5_ constituents, especially secondary inorganic aerosols such as nitrate, sulfate, and ammonium, resulting in a greater-than-additive increase in stroke mortality (49).

Interesting, the HRs for black carbon, organic matter, and sulfate increased after full adjustment while those for nitrate and ammonium remained stable or slightly decreased. This differential pattern likely reflects negative confounding driven by urban–rural socioeconomic gradients. Carbonaceous constituents are primarily emitted from traffic and combustion sources, which are concentrated in urban areas where residents tend to have better healthcare access, higher socioeconomic status, and healthier behaviors that lower stroke risk. Adjusting for these variables removed their masking effect, thereby unmasking the true associations.

Stratified analyses showed significantly stronger associations among older adults (≥ 65 years), a finding consistent with previous epidemiological evidence (11, 50, 51). This may be attributed to age-related physiological degradation, including cardiovascular aging and weakened immune function, which can amplify vulnerability to the cerebrovascular injury induced by PM_2.5_ constituents (52). Additionally, the progressive accumulation of cardiovascular risk factors over the life course, such as hypertension, diabetes, dyslipidemia, vascular stiffness, and endothelial dysfunction, may further exacerbate this susceptibility (53). Notably, many of these conditions are themselves influenced by exposure to PM_2.5_ constituents, suggesting a potential synergistic pathway in the development of stroke (54–57). Another notable finding was the elevated susceptibility among residents in southern China, a pattern consistent with existing evidence. A national screening program identified the highest carotid plaque prevalence in southwestern China (43.17%), a known precursor to stroke (58). Two recent studies on cardiovascular diseases and PM_2.5_ constituents also identified stronger adverse effects in southern populations (32, 59). This north–south disparity likely arises from a combination of factors, including climatic conditions that promote secondary aerosol formation in the south, alongside differences in emission sources, pollution levels, and population characteristics.

In light of our results, targeted strategies are warranted to mitigate stroke risk from PM_2.5_ constituents. At the policy level, transitioning to a clean energy economy and phasing out industrial animal farming are critical steps (60, 61). For vulnerable individuals, practical measures such as using air filtration systems, wearing protective masks outdoors, and scheduling activities around air quality forecasts are recommended (62). Furthermore, expanding urban green infrastructure can serve as an effective public health intervention to ameliorate the cardiovascular burden of air pollution (63).

Our study possesses several key strengths. First, the prospective cohort study design with a nationally representative sample ensured the reliability of our conclusions, while a series of sensitivity analyses further confirms the stability and robustness of the observed associations. Second, environmental exposure data were derived from a model with high spatial and temporal resolution, thereby reducing exposure misclassification in comparison with the use of fixed-site monitoring stations alone. Third, the application of the QGC model enabled the examination of the individual contribution of each constituent, effectively addressing the collinearity inherent in mixtures of pollutants. However, several limitations in our study should be acknowledged. First, while our sample size is nationally representative and provided sufficient statistical power to detect robust associations (as evidenced by narrow confidence intervals), it is more modest than some studies utilizing large administrative databases. However, the major strength of CHARLS cohort lies in its rich, individual-level data on a wide array of potential confounders, enabling more rigorous confounding control. Second, exposure was estimated at the city level rather than at individual residential addresses due to the privacy protections of the CHARLS public-use dataset. While city-level aggregation is a common approach in CHARLS-based environmental health studies (64), it inevitably introduces non-differential exposure misclassification, particularly for black carbon, given its strong within-city spatial heterogeneity from traffic and combustion sources. Such misclassification typically biases estimates toward the null, implying our findings, especially for black carbon, are likely conservative and the true risks may be even greater. Future studies with access to geocoded residential addresses or incorporate automatic and portable monitoring to improve the accuracy of individual exposure assessment are warranted to validate our findings. Third, although we adjusted for a range of individual-level covariates, residual confounding remains possible. Several unmeasured factors may influence both PM_2.5_ constituent exposure and incident stroke risk, including socioeconomic characteristics, healthcare access, occupational exposures, dietary factors, and co-pollutant exposures. Importantly, given the robust associations observed even after extensive adjustment, it is unlikely that residual confounding alone can fully explain our findings. Nonetheless, this limitation should be considered when interpreting the observed associations. Additionally, both the QGC and WQS models provide fixed weights for mixture constituents without confidence intervals, limiting the ability to evaluate the statistical significance of these weights. Fourth, incident stroke was ascertained through self-reported physician diagnoses rather than medical record validation. Prior validation studies using CHARLS data have shown that self-reported chronic conditions consistently demonstrate high specificity when compared with biomedical measurements (65, 66). Nonetheless, some degree of outcome misclassification cannot be entirely ruled out. Specifically, it is possible that some stroke cases were underreported by participants, and we could not determine whether fatal strokes were included unless they were captured in the last available interview before death. Additionally, the stroke cases in the CHARLS survey were not classified into subtypes. Therefore, our findings reflect the overall association between PM_2.5_ constituents and incident stroke, without distinguishing which subtype may be more severely affected. Furthermore, participants without a stroke event were censored at the date of their 2018 interview, which varied across individuals. Future studies with access to validated, subtype-specific stroke data are warranted to confirm and extend our findings. Fifth, residential mobility during follow-up was not accounted for in our exposure assessment. While the CHARLS survey collects longitudinal data, publicly available individual-level information on address changes between waves is limited, and the follow-up intervals preclude determining the exact timing of residential relocations. Consequently, we were unable to update exposure estimates for participants who may have moved during the follow-up period. Future studies with geocoded residential histories or continuous address tracking are warranted to refine exposure assessment and validate our findings. Finally, selection bias is difficult to avoid when conducting inclusion and exclusion, as participants excluded due to missing data or loss to follow-up differed from those included in some demographic characteristics (Supplementary Table S1). However, the differences in key cardiovascular risk factors (such as hypertension and diabetes prevalence) were minimal, suggesting that the potential impact of selection bias on our effect estimates is likely limited. Nonetheless, the generalizability of our findings to other populations or regions with different pollution profiles warrants further investigation.

## 5 Conclusion

In summary, this prospective cohort study provides robust evidence linking long-term exposure to specific PM_2.5_ constituents with an increased risk of incident stroke among middle-aged and older adults in China. Our findings underscore the predominant role of carbonaceous components, particularly black carbon and organic matter, and highlight the heightened susceptibility of older adults and residents of southern China. Given the rapidly aging population and the distinct north–south environmental and demographic profiles in China, these results hold significant implications for guiding age- and region-specific clinical preventive strategies, public health interventions, and targeted environmental policies to mitigate the stroke burden.

## Data Availability

The original contributions presented in the study are included in the article/Supplementary material, further inquiries can be directed to the corresponding author.

## Footnotes

1
http://charls.pku.edu.cn/

2
http://tapdata.org.cn/

3
https://cds.climate.copernicus.eu/
