# Supplementary material for: Long-term exposure to fine particulate matter constituents and incident stroke risk among middle-aged and older adults in China: a cohort study
Source: Front Public Health. 2026 Sep 9;14:1909901. doi: 10.3389/fpubh.2026.1909901 (PMC13597756; doi:10.3389/fpubh.2026.1909901)
Supplement: Supplementary file 1 [file Data_Sheet_1.docx]

Supplementary Material

Long-term exposure to fine particulate matter constituents and incident stroke risk among middle-aged and older adults in China: A cohort study

Hao Wang^1^, Miaomiao Wei^1^, Taikun Lu^4^, Chao Wang^2^, Xiaoshuang Xia^1^, Lin Wang^2^, Xin Li^1,2,3,*^

^1^ Department of Neurology, the Second Hospital of Tianjin Medical University, Tianjin, 300211, China

^2^ Department of Geriatrics, the Second Hospital of Tianjin Medical University, Tianjin, 300211, China

^3^ Tianjin Health Meteorological Cross Innovation Center, Tianjin, 300211, China

^4^ Department of Neurosurgery, Peking Union Medical College Hospital, Chinese Academy of Medical Science and Peking Union Medical College, Beijing, 100730, China

^*^Corresponding author: Xin Li, Department of Neurology, Second Hospital of Tianjin Medical University, No. 23, Pingjiang Road, Hexi District, Tianjin, 300211, China.

E-mail: [xinli02@tmu.edu.cn](mailto:xinli02@tmu.edu.cn); Tel: 13902082748.

# 1 Supplementary Data

Supplementary Table S1. Comparison of baseline characteristics between participants included in the final analysis and those excluded due to missing data or loss to follow-up.

Supplementary Table S2. Distributions of PM_2.5_ and its constituents among rural residents.

Supplementary Table S3 Distributions of PM_2.5_ and its constituents among urban residents.

Supplementary Table S4. Characteristics of exposure-response relationships between PM_2.5_ and its constituents and the risk of incident stroke.

Supplementary Table S5. Residual-based analysis of PM_2.5_ constituent toxicity.

Supplementary Table S6. Sensitivity analysis by extreme value exclusion.

Supplementary Table S7. Proportional hazards assumption testing and stratified Cox model results.

Supplementary Table S8. Gender-stratified results from the Cox proportional hazards models.

Supplementary Figure S1. Spatial distributions of PM_2.5_ and its constituents across China during the study period.

Supplementary Figure S2. Pearson correlation coefficients between PM_2.5_ and its constituents.

Supplementary Figure S3. Variance Inflation Factor values for PM_2.5_ and its constituents.

Supplementary Figure S4. Weights of PM_2.5_ constituents in the mixture index for the association between the particle mixture and incident stroke by WQS.

Supplementary Figure S5. Adjusted hazard ratio (HR) (95% confidence interval [CI]) of the risk of incident stroke by PM_2.5_ and its constituents.

# 2 Supplementary Tables and Figures

## 2.1 Supplementary Tables

**Supplementary Table S1** Comparison of baseline characteristics between participants included in the final analysis and those excluded due to missing data or loss to follow-up.

| Characteristics | Included  (n=8724) | Excluded  (n=9881) | *p*-value |
| --- | --- | --- | --- |
| Age (years) | 59.95 ± 8.86 | 59.93 ± 11.36 | 0.913 |
| Gender, n (%) |  |  | < 0.001 |
| Female | 4435 (50.80) | 5298 (53.70) |  |
| Male | 4289 (49.20) | 4572 (46.30) |  |
| Education, n (%) |  |  | < 0.001 |
| Primary school or below | 7792 (89.30) | 8367 (84.70) |  |
| High school | 799 (9.20) | 1185 (12.00) |  |
| College or above | 133 (1.50) | 329 (3.30) |  |
| Marital status, n (%) |  |  | < 0.001 |
| Married | 7727 (88.60) | 8409 (85.10) |  |
| Unmarried | 997 (11.40) | 1472 (14.90) |  |
| Residence, n (%) |  |  |  |
| Rural | 7145 (81.90) | 7044 (71.70) | < 0.001 |
| Urban | 1579 (18.10) | 2783 (28.30) |  |
| Smoking, n (%) |  |  | < 0.001 |
| No | 4793 (54.90) | 3695 (48.90) |  |
| Yes | 3931 (45.10) | 3864(51.10) |  |
| Drinking, n (%) |  |  | < 0.001 |
| No | 4665 (53.50) | 5438(56.50) |  |
| Yes | 4059 (46.50) | 4193 (43.50) |  |
| Sleep time (hours) | 6.18 ± 1.82 | 6.24 ± 1.87 | 0.019 |
| Social activity, n (%) |  |  | < 0.001 |
| No | 3555 (40.70) | 3495 (35.40) |  |
| Yes | 5169 (59.30) | 6386 (64.60) |  |
| Hypertension, n (%) |  |  | 0.132 |
| No | 6404 (73.40) | 7155 (72.40) |  |
| Yes | 2320 (26.60) | 2726 (27.60) |  |
| Dyslipidemia, n (%) |  |  | 0.133 |
| No | 7725 (88.50) | 8678 (87.80) |  |
| Yes | 999 (11.50) | 1203 (12.20) |  |
| Diabetes, n (%) |  |  | 0.059 |
| No | 8117 (93.00) | 9121 (92.30) |  |
| Yes | 607 (7.00) | 760 (7.70) |  |
| Heart disease, n (%) |  |  | 0.053 |
| No | 7588 (87.00) | 8497 (86.00) |  |
| Yes | 1136 (13.00) | 1384 (14.00) |  |
| SBP (mmHg) | 129.89 ± 20.72 | 131.89 ± 22.73 | < 0.001 |
| DBP (mmHg) | 76.48 ± 13.11 | 77.79 ± 14.04 | < 0.001 |
| BMI (kg/m^2^) | 23.84 ± 3.77 | 23.90 ± 3.96 | 0.411 |
| WC (cm) | 86.71 ± 10.91 | 87.02 ± 11.26 | 0.137 |

SBP, systolic blood pressure; DBP, diastolic blood pressure; BMI, body mass index; WC, waist circumference.

**Supplementary Table S2** Distributions of PM_2.5_ and its constituents among rural residents.

| Variables | Mean | SD | Minimum | P_25_ | Median | P_75_ | Maximum |
| --- | --- | --- | --- | --- | --- | --- | --- |
| PM_2.5_ (μg/m^3^) | 50.7 | 18.6 | 17.2 | 34.6 | 49.1 | 63.3 | 118.1 |
| Sulfate (μg/m^3^) | 9.4 | 3.0 | 3.4 | 6.6 | 9.4 | 11.8 | 19.7 |
| Nitrate (μg/m^3^) | 10.8 | 4.9 | 2.5 | 6.1 | 11.0 | 15.0 | 26.5 |
| Ammonium (μg/m^3^) | 7.3 | 3.0 | 2.3 | 4.8 | 7.5 | 9.5 | 16.7 |
| Organic matter (μg/m^3^) | 12.4 | 4.2 | 4.5 | 9.1 | 11.9 | 15.0 | 35.7 |
| Black carbon (μg/m^3^) | 2.4 | 0.7 | 1.1 | 1.9 | 2.3 | 3.0 | 5.7 |

SD, standard deviation; P_25_, 25th percentile (first quartile); P_75_, 75th percentile (third quartile); PM_2.5_, particulate matter with aerodynamic diameter ≤ 2.5 μm.

**Supplementary Table S3** Distributions of PM_2.5_ and its constituents among urban residents.

| Variables | Mean | SD | Minimum | P_25_ | Median | P_75_ | Maximum |
| --- | --- | --- | --- | --- | --- | --- | --- |
| PM_2.5_ (μg/m^3^) | 51.0 | 17.0 | 18.1 | 38.8 | 49.0 | 61.0 | 103.6 |
| Sulfate (μg/m^3^) | 9.3 | 2.9 | 3.7 | 7.2 | 9.1 | 11.6 | 17.7 |
| Nitrate (μg/m^3^) | 10.7 | 4.6 | 2.5 | 7.2 | 10.3 | 13.8 | 25.7 |
| Ammonium (μg/m^3^) | 7.2 | 2.8 | 2.3 | 5.2 | 6.7 | 9.2 | 16.1 |
| Organic matter (μg/m^3^) | 12.3 | 3.8 | 4.5 | 9.9 | 11.6 | 14.5 | 25.3 |
| Black carbon (μg/m^3^) | 2.4 | 0.6 | 1.1 | 2.0 | 2.3 | 2.9 | 4.7 |

SD, standard deviation; P_25_, 25th percentile (first quartile); P_75_, 75th percentile (third quartile); PM_2.5_, particulate matter with aerodynamic diameter ≤ 2.5 μm.

**Supplementary Table S4** Characteristics of exposure-response relationships between PM_2.5_ and its constituents and the risk of incident stroke.

|  | Median (μg/m^3^) | Max HR | Concentration at Max HR (μg/m^3^) | Threshold (μg/m^3^) | HR at Threshold |
| --- | --- | --- | --- | --- | --- |
| PM_2.5_ | 49.1 | 3.33 | 85.2 | 60.6 | 1.034 |
| Sulfate | 9.3 | 3.35 | 14.3 | 10.5 | 0.887 |
| Nitrate | 11.0 | 2.22 | 19.4 | 12.4 | 0.943 |
| Ammonium | 7.5 | 2.35 | 12.3 | 8.3 | 0.935 |
| Organic matter | 11.8 | 3.05 | 19.7 | 14.3 | 1.077 |
| Black carbon | 2.3 | 4.04 | 3.6 | 2.8 | 1.062 |

HR, hazard ratio; AUC, area under the curve; PM_2.5_, particulate matter with aerodynamic diameter ≤ 2.5 μm.

**Supplementary Table S5** Residual-based analysis of PM_2.5_ constituent toxicity.

| Pollutants | HR (95% CI) ^a^ | *p*-value | Weight | Rank |
| --- | --- | --- | --- | --- |
| Black carbon | 6.84 (3.95-12.15) | < 0.001 | 0.810 | 1 |
| Organic matter | 2.68 (1.72-4.29) | < 0.001 | 0.232 | 2 |
| Sulfate | 1.71 (0.76-3.96) | 0.161 | 0.098 | 3 |
| Ammonium | 0.69 (0.44-1.13) | 0.097 | -0.043 ^b^ | 4 |
| Nitrate | 0.30 (0.18-0.51) | < 0.001 | -0.096 ^b^ | 5 |

HR, hazard ratio; CI, confidence interval; PM_2.5_, particulate matter with aerodynamic diameter ≤ 2.5 μm.

^a^ Based on Cox proportional hazards models applied to residual values. Residuals represent constituent-specific variation independent of total PM_2.5_ mass. All models adjusted for age, gender, education, marital status, residence, smoking, drinking, sleep time, social activity, hypertension, dyslipidemia, diabetes, heart disease, body mass index, waist circumference, temperature, and relative humidity.

^b^ Negative weights indicate a protective or inverse association with the outcome.

**Supplementary Table S6** Sensitivity analysis by extreme value exclusion.

| Pollutants | Main Model ^a^  HR (95% CI) | *p*-value | Extreme Exclusion ^b^  HR (95% CI) | *p*-value |
| --- | --- | --- | --- | --- |
| PM_2.5_ | 1.55 (1.35-1.79) | < 0.001 | 1.15 (1.00-1.33) | 0.054 |
| Sulfate | 1.55 (1.34-1.79) | < 0.001 | 1.13 (0.96-1.34) | 0.146 |
| Nitrate | 1.37 (1.18-1.59) | < 0.001 | 1.14 (0.97-1.34) | 0.108 |
| Ammonium | 1.42 (1.24-1.63) | < 0.001 | 1.16 (0.99-1.35) | 0.062 |
| Organic matter | 1.59 (1.40-1.80) | < 0.001 | 1.11 (0.97-1.26) | 0.135 |
| Black carbon | 1.77 (1.54-2.03) | < 0.001 | 1.14 (0.99-1.32) | 0.065 |

HR, hazard ratio; CI, confidence interval; PM_2.5_, particulate matter with aerodynamic diameter ≤ 2.5 μm.

^a^ Main Model: Cox proportional hazards models were adjusted for age, gender, education, marital status, residence, smoking, drinking, sleep time, social activity, hypertension, dyslipidemia, diabetes, heart disease, body mass index, waist circumference, temperature, and relative humidity.

^b^ Extreme Exclusion: The same Cox proportional hazards models as in the main analysis were refitted after excluding extreme values (the top and bottom 1% of each pollutant's distribution), using identical covariate adjustments.

**Supplementary Table S7** Proportional hazards assumption testing and stratified Cox model results.

| Pollutants | Proportional Hazards Assumption Violation ^a^ | Global Test  *p*-value | HR (95% CI) ^b^ | *p*-value |
| --- | --- | --- | --- | --- |
| PM_2.5_ | Yes | < 0.001 | 1.55 (1.35-1.79) | < 0.001 |
| Sulfate | Yes | < 0.001 | 1.55 (1.34-1.79) | < 0.001 |
| Nitrate | Yes | < 0.001 | 1.37 (1.18-1.59) | < 0.001 |
| Ammonium | Yes | < 0.001 | 1.42 (1.24-1.63) | < 0.001 |
| Organic matter | Yes | < 0.001 | 1.58 (1.40-1.80) | < 0.001 |
| Black carbon | Yes | < 0.001 | 1.77 (1.54-2.04) | < 0.001 |

HR, hazard ratio; CI, confidence interval; PM_2.5_, particulate matter with aerodynamic diameter ≤ 2.5 μm.

^a^ Proportional Hazards Assumption Violation: Based on Schoenfeld residuals global test (*p* < 0.05 indicates violation of the proportional hazards assumption).

^b^ The hazard ratios (HR) (95% confidence interval [CI]) are identical between the main Cox models and the gender-stratified Cox models. Main models were adjusted for age, gender, education, marital status, residence, smoking, drinking, sleep time, social activity, hypertension, dyslipidemia, diabetes, heart disease, body mass index, waist circumference, temperature, and relative humidity. Stratified Cox models used the same adjustments but were stratified by gender instead of including it as a covariate.

**Supplementary Table S8** Gender-stratified results from the Cox proportional hazards models.

| Pollutants | Female | |  | Male | |
| --- | --- | --- | --- | --- | --- |
|  | HR (95% CI) ^a^ | $p$-value |  | HR (95% CI) ^a^ | $p$-value |
| PM_2.5_ | 1.58 (1.28-1.94) | < 0.001 |  | 1.53 (1.26-1.86) | < 0.001 |
| Sulfate | 1.61 (1.29-1.99) | < 0.001 |  | 1.50 (1.23-1.84) | < 0.001 |
| Nitrate | 1.43 (1.15-1.77) | 0.001 |  | 1.32 (1.08-1.62) | 0.007 |
| Ammonium | 1.48 (1.21-1.81) | < 0.001 |  | 1.37 (1.13-1.66) | 0.001 |
| Organic matter | 1.53 (1.27-1.84) | < 0.001 |  | 1.63 (1.37-1.94) | < 0.001 |
| Black carbon | 1.69 (1.38-2.06) | < 0.001 |  | 1.86 (1.53-2.25) | < 0.001 |

HR, hazard ratio; CI, confidence interval; PM_2.5_, particulate matter with aerodynamic diameter ≤ 2.5 μm.

^a^ Cox proportional hazards models were adjusted for age, education, marital status, residence, smoking, drinking, sleep time, social activity, hypertension, dyslipidemia, diabetes, heart disease, body mass index, waist circumference, temperature, and relative humidity.

## 2.2 Supplementary Figures

**Supplementary Figure 1** Spatial distributions of PM_2.5_ and its constituents across China during the study period. PM_2.5_, particulate matter with aerodynamic diameter ≤ 2.5 μm.

**Supplementary** **Figure S2** Pearson correlation coefficients between PM_2.5_ and its constituents. PM_2.5_, particulate matter with aerodynamic diameter ≤ 2.5 μm.

**Supplementary** **Figure S3** Variance Inflation Factor values for PM_2.5_ and its constituents. VIF, variance inflation factors; PM_2.5_, particulate matter with aerodynamic diameter ≤ 2.5 μm.

**Supplementary Figure S4** Weights of PM_2.5_ constituents in the mixture index for the association between the particle mixture and incident stroke by WQS. The model was adjusted for age, gender, education, marital status, residence, smoking, drinking, sleep time, social activity, hypertension, dyslipidemia, diabetes, heart disease, body mass index, waist circumference, temperature, and relative humidity. PM_2.5_, particulate matter with aerodynamic diameter ≤ 2.5 μm; WQS, weighted quantile sum.

**Supplementary Figure S5** Adjusted hazard ratio (HR) (95% confidence interval [CI]) of the risk of incident stroke by PM_2.5_ and its constituents. HR (95% CI) of stroke associated with per interquartile range increase of PM_2.5_ constituents from single-pollutant Cox models. Model 1 did not incorporate any covariates. Model 2 was adjusted for age, gender, education, marital status and residence. Model 3 was further adjusted for smoking, drinking, sleep time, social activity, hypertension, dyslipidemia, diabetes, heart disease, body mass index, waist circumference, temperature, and relative humidity. HR, hazard ratio; CI, confidence interval; PM_2.5_, particulate matter with aerodynamic diameter ≤ 2.5 μm.
